# A three-lncRNA signature predicts clinical outcomes in low-grade glioma patients after radiotherapy

**DOI:** 10.18632/aging.103189

**Published:** 2020-05-26

**Authors:** Wanzun Lin, Zongwei Huang, Yanyan Xu, Xiaochuan Chen, Ting Chen, Yuling Ye, Jianming Ding, Zhangjie Chen, Long Chen, Xianxin Qiu, Sufang Qiu

**Affiliations:** 1 Department of Radiation Oncology, Fujian Cancer Hospital and Fujian Medical University Cancer Hospital, Fuzhou, China; 2 Department of Radiation Oncology, Fujian Medical University Cancer Hospital and Fujian Cancer Hospital, Fuzhou, China; 3 Department of Obstetrics and Gynecology, Shanghai General Hospital, Shanghai Jiao Tong University School of Medicine, Shanghai, China; 4 Department of Chemotherapy, The First Affiliated Hospital of Fujian Medical University, Fuzhou, China; 5 Division of Neurocritical Care, Huashan Hospital, Fudan University, Shanghai, China; 6 Department of Radiation Oncology, Shanghai Proton and Heavy Ion Center, Shanghai, China

**Keywords:** long non-coding RNAs, radiosensitivity, low-grade glioma, prognosis, bioinformatic analysis

## Abstract

Although radiation therapy (RT) plays a critical role in the treatment of low-grade glioma (LGG), many patients suffer from adverse effects without experiencing survival benefits. In various carcinomas, long non-coding RNAs (lncRNAs) contribute to pathogenic processes, including tumorigenesis, metastasis, chemoresistance, and radioresistance. Currently, the role of lncRNAs in the radiosensitivity of LGG is largely unknown. Here, we downloaded clinical data for 167 LGG patients from The Cancer Genome Atlas database and divided them between radiosensitive and radioresistant groups based on their clinical outcomes after receiving radiotherapy. We identified 37 lncRNAs that were differentially expressed (DElncRNAs) between the groups. Functional enrichment analysis revealed that their potential target mRNAs were mainly enriched in the PI3K-Akt and MAPK signaling pathways and in DNA damage response. Kaplan-Meier survival analysis revealed that increased expression of six lncRNAs was significantly associated with radiosensitivity. We then developed a risk signature based on three of the DElncRNAs that served as an independent biomarker for predicting LGG patient outcomes after radiotherapy. In vitro experiments further validated the biological function of these lncRNAs on low-grade glioma radiation response.

## INTRODUCTION

Low-grade glioma (LGG) accounts for 15–20% of all glioma cases and is associated with median survival times of at least 10 years [1]. Tumor volume/stage, genetic features, and treatment response are the major prognostic factors, and surgery and radiation therapy (RT) are the most common treatments for LGG [2, 3]. However, after RT, some patients suffer from acute side effects (e.g., fatigue, anorexia, nausea, headache, and insomnia) and delayed radiation injuries (e.g., cognitive impairment and endocrine dysfunction) without experiencing survival benefits [1]. A model for predicting radiosensitivity would help improve treatment efficacy and quality of life in LGG patients by reducing harmful side-effects.

Recently, novel lncRNAs have been identified as significant biomarkers for cancer prognosis, diagnosis, and prediction of therapeutic outcomes [4]. LncRNAs are pervasive transcripts greater than 200 nucleotides in length with little or no protein-coding ability [5]. Dysregulation of lncRNAs, which play vital regulatory roles in cellular pathophysiological processes (e.g. proliferation, invasion, apoptosis, metastasis, drug resistance, and radioresistance), is associated with tumorigenesis [6–9]. However, the roles and prognostic values of lncRNAs in LGG radiotherapy are largely unknown.

In this study, we downloaded radiotherapeutic response information for 167 LGG patients from The Cancer Genome Atlas database. The patients were divided into two clusters based on their radiotherapy outcomes: the radiosensitive group consisted of those who demonstrated complete or partial responses, while the radioresistant group had stable or radiographic progressive disease. Differentially expressed lncRNAs (DElncRNAs) associated with radiation response in LGG patients were identified and correlations between these DElncRNAs and overall survival (OS) and progression-free survival (PFS) were examined. A three-lncRNA signature was constructed and served as an independent prognostic indicator for OS in LGG patients that received radiotherapy. Additionally, underlying mechanisms associated with the three-lncRNA signature in predicting the outcome of radiotherapy were further elucidated.

## RESULTS

### Identification of differentially expressed lncRNAs associated with radiation response

A total of 167 LGG patients for which radiation response information was available were downloaded in this study. The clinicopathological and molecular characteristics of these patients are shown in Table 1. Patients with complete response (CR) or partial response (PR) after radiotherapy were assigned to the radiosensitive group, while those with stable disease (SD) or radiographic progressive disease (PD) were assigned to the radioresistant group.

**Table 1:** Clinical characteristic.

**Clinical characteristics**	**Total (N=167)**	
	**N**	**%**
Age		
<45	86	51.4
≥45	81	48.6
Gender		
Female	81	48.6
Male	86	51.4
Radiation response		
Complete response	32	19.2
Partial response	11	6.6
Stable disease	103	61.7
Radiographic progressive disease	21	12.5
Histological type		
Astrocytoma	85	50.9
Oligoastrocytoma	36	21.6
Oligodendroglioma	46	27.5
Grade		
G2	52	31.1
G3	115	68.9

**Figure 1:**
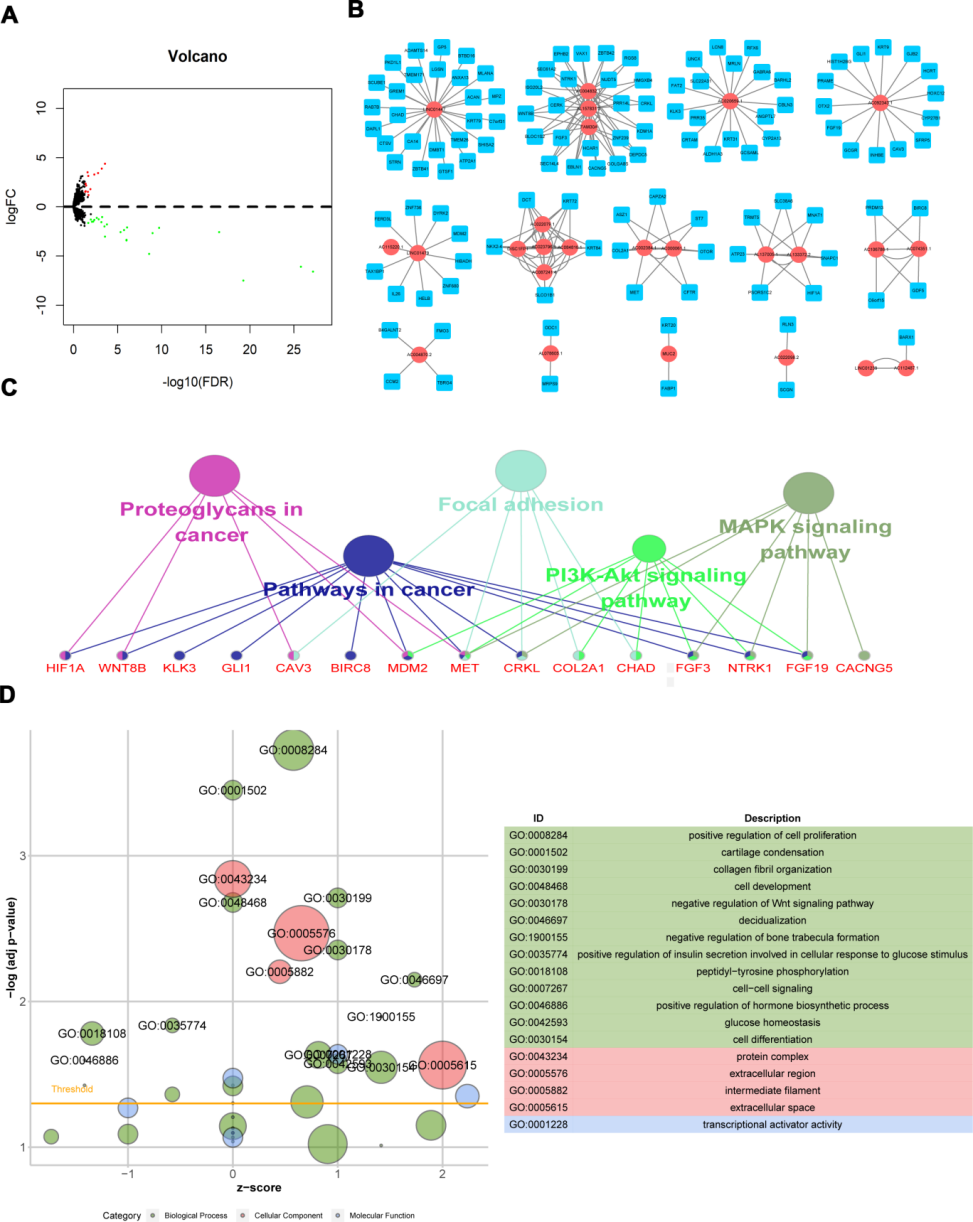
**Differentially expressed lncRNAs associated with radiation response and biological functions of their potential target mRNAs.** (**A**) Volcano map of DElncRNAs; (**B**) Radiosensitivity-related lncRNA-mRNA regulator network; (**C**) KEGG pathway of genes co-expressed with DElncRNAs; (**D**) GO functions of genes co-expressed with DElncRNAs.

LncRNA expression was analyzed in 124 radioresistant and 43 radiosensitive samples. Using the “edgeR” package in R software, 37 DElncRNAs were identified from the expression profile using thresholds of *p*<0.05 and |log_2_ fold change|>1; 13 lncRNAs were up-regulated, and 24 were down-regulated, in the radioresistant group (Supplementary 1). The distribution of all DElncRNAs in the -log (FDR) and logFC dimensions are shown in a volcano map in Figure 1A.

### Radiosensitivity-related lncRNA-mRNA regulator network and the biological function of genes co-expressed with DElncRNAs

The 37 DElncRNAs were used as bait to identify regulatory mRNAs in a weighted correlation network analysis (WGCNA). Edge weights > 0.5 were calculated and used to construct lncRNA-mRNA regulatory networks in which higher values indicated a stronger connection between or co-expression of genes. The co-expression network was composed of 148 nodes and 229 connections between 25 DElncRNAs and 123 mRNAs (Figure 1B). Among the 148 nodes, 8 central node genes with more than 10 connections each that might play crucial roles in radiosensitvity were identified: LINC01447, AC004832.1, AC020659.1, AC087241.4, AC092343.1, AL157831.2, DISC1FP1, and FAM30A.

To elucidate the functions and signaling pathways associated with genes co-expressed with DElncRNAs, Gene Ontology (GO) and Kyoto Encyclopedia of Genes and Genomes (KEGG) enrichment analyses were conducted. The KEGG analysis showed that co-expressed genes were mainly enriched in cancer pathways, including the PI3K-Akt and MAPK signaling pathways (Figure 1C). GO functions analysis showed that co-expressed genes mainly activated cell proliferation and DNA damage response and inhibited apoptotic processes, which might contribute to radioresistance (Figure 1D).

### Prognostic value of DElncRNAs in LGG patients receiving radiotherapy

**Figure 2:**
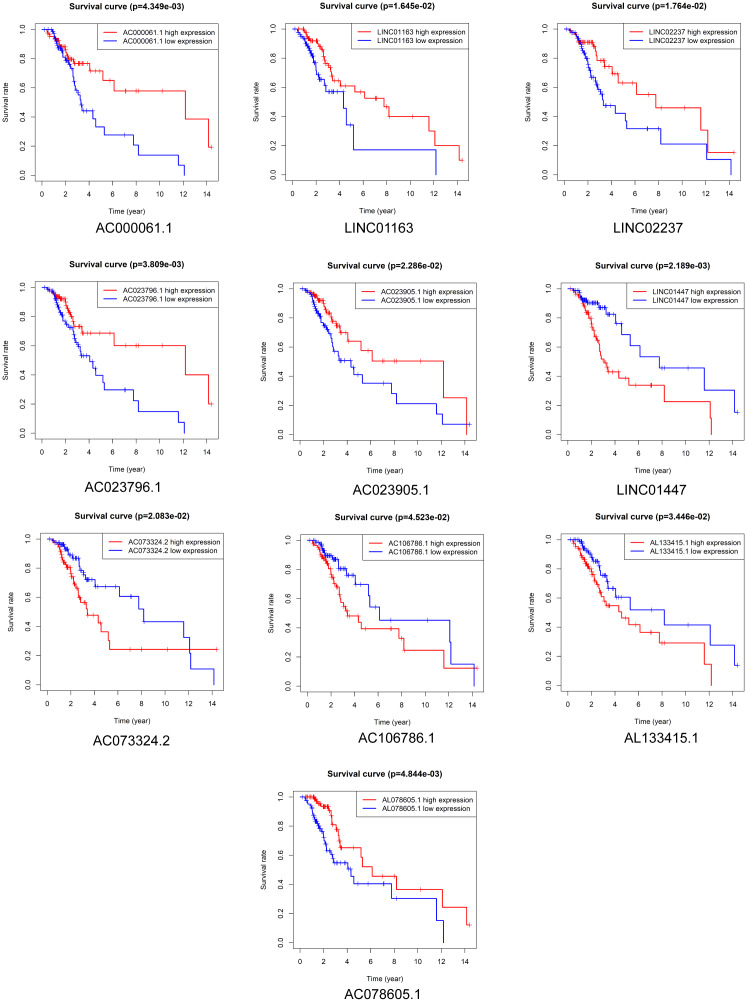
**Prognostic value of DElncRNAs in predicting LGG patient OS after radiotherapy.**

**Figure 3:**
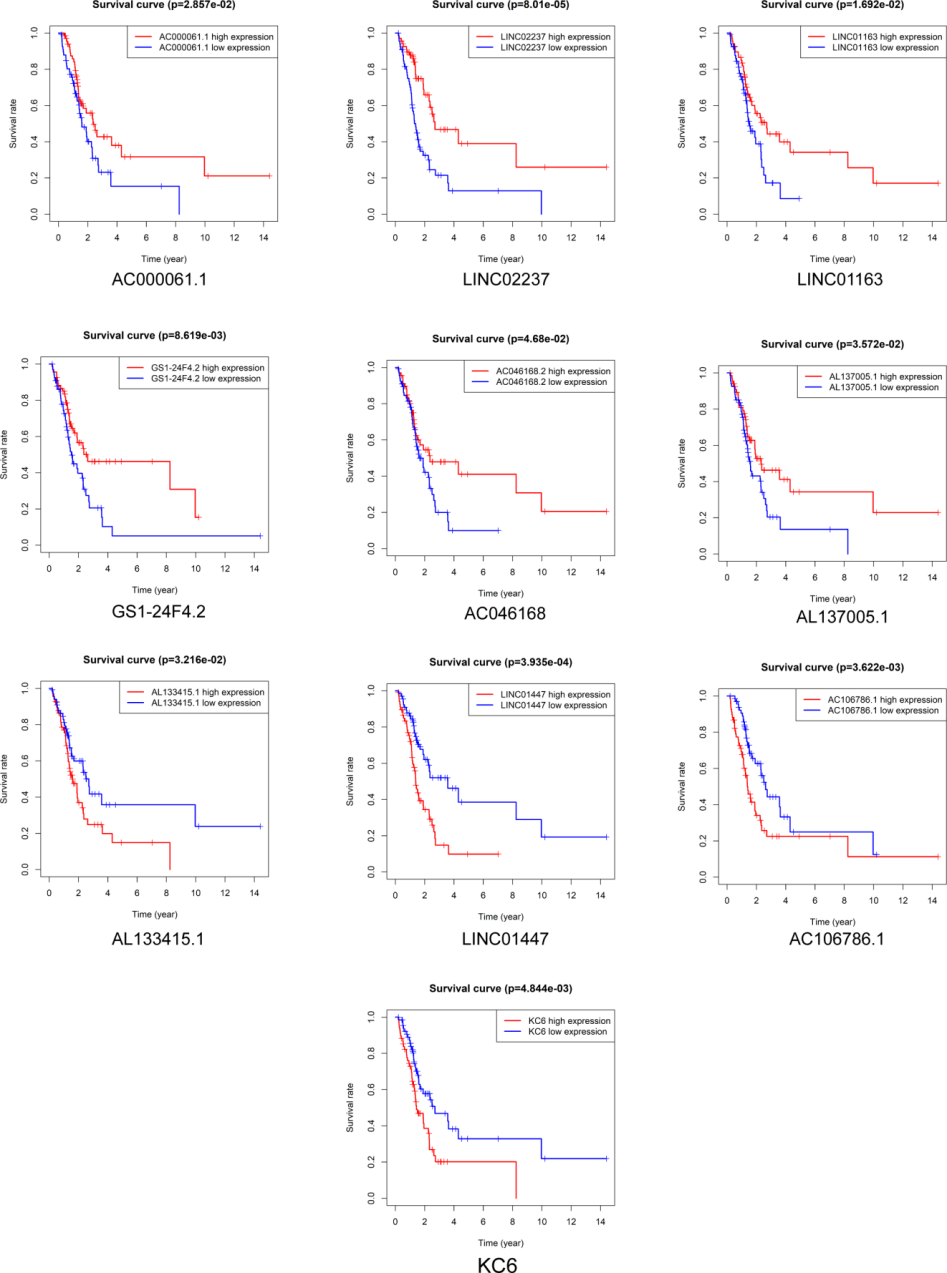
**Prognostic value of DElncRNAs in predicting LGG patient PFS after radiotherapy.**

Kaplan-Meier survival analysis was performed to evaluate the prognostic value of the 37 DElncRNAs in LGG patients who received radiotherapy. In total, 10 lncRNAs were significantly associated with OS: LINC01447, AC023796.1, AC000061.1, AL078605.1, LINC01163, LINC02237, AC073324.2, AC023905.1, AL133415.1, and AC106786.1 (Figure 2). Ten lncRNAs were significantly associated with PFS: LINC01447, LINC02237, AC106786.1, KC6, GS1-24F4.2, LINC01163, AC000061.1, AL133415.1, AL137005.1, and AC046168.2 (Figure 3).

Notably, high expression of AL133415.1, LINC01447, and AC106786.1 predicted poor prognosis after radiotherapy as indicated by reduced OS and PFS; these lncRNAs might therefore be risk factors. High expression of AC000061.1, LINC01163, and LINC02237 predicted good prognosis after radiotherapy as indicated by longer OS and PFS; these lncRNAs might therefore be protective factors.

### Three-lncRNA signature as a prognostic risk model for LGG patients after radiotherapy

**Figure 4:**
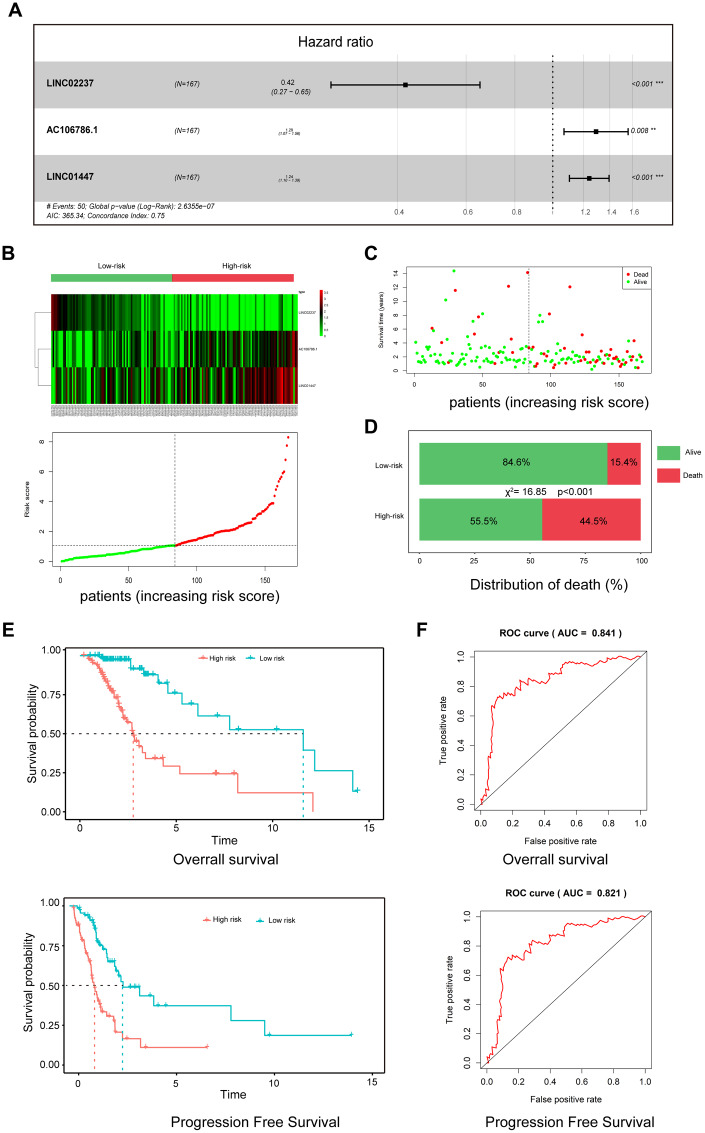
**A three-lncRNA signature predicts LGG patient prognosis.** (**A**) Construction of risk model by multivariate Cox regression; (**B**) Heatmap of seven lncRNA expression profiles and distribution of seven associated lncRNA-based risk scores; (**C**) Distributions showing patient status in high- and low-risk groups; (**D**) Mortality rates in high- and low-risk groups. (**E**) Survival curves of patients assigned to high- and low-risk groups; (**F**) ROC curves showing the predictive efficiency of the risk signature on survival.

To establish a risk model for predicting prognosis of LGG patients after radiotherapy, univariate and multivariate Cox regression analysis were performed using 37 DElncRNAs. In the univariate analysis, 10 DElncRNAs were significantly associated with OS. In the multivariate analysis, three lncRNAs with *P*<0.01 (AC106786.1, LINC02237, and LINC01447) were included in the predictive model (Figure 4A). A risk score was calculated using the following formula: risk score = 0.22×LINC01447 + 0.26×AC106786.1 − 0.86×LINC02237.

As shown in the heatmap in Figure 1B, expression of LINC01447 and AC106786.1 were increased in patients with higher risk scores, while LINC02237 expression was increased in those with lower risk scores. Associations between risk score and cancer-related death were calculated (Figure 4C); the results indicated that the mortality rate in high-risk group was significantly higher than that in the low-risk group (Figure 4D). Next, we explored whether the three-lncRNA signature was associated with OS and PFS in patients who received radiotherapy; patients who had high risk scores tended to have shorter OS and PFS times after radiotherapy treatment (Figure 4E). ROC curve analysis was performed to validate the accuracy of the three-lncRNA signature in predicting patients’ susceptibility to radiotherapy. The AUC values for OS and PFS were 0.841 and 0.821, indicating that the risk prediction model had high sensitivity and specificity (Figure 4F).

We further defined the threshold for risk score. In our research, the standards for high and low risk scores were evaluated on the basis of cut points associated with the Youden Index (derived from the AUROC for survival). Cut-off values of 1.952 for the risk model was defined, which served to divide the patients into a high risk group (with levels of score ≥ 1.952) and a low risk group (with levels of risk score < 1.952).

### Prognostic risk model was associated with clinicopathological features and served as an independent prognostic indicator among LGG patients after radiotherapy

**Figure 5:**
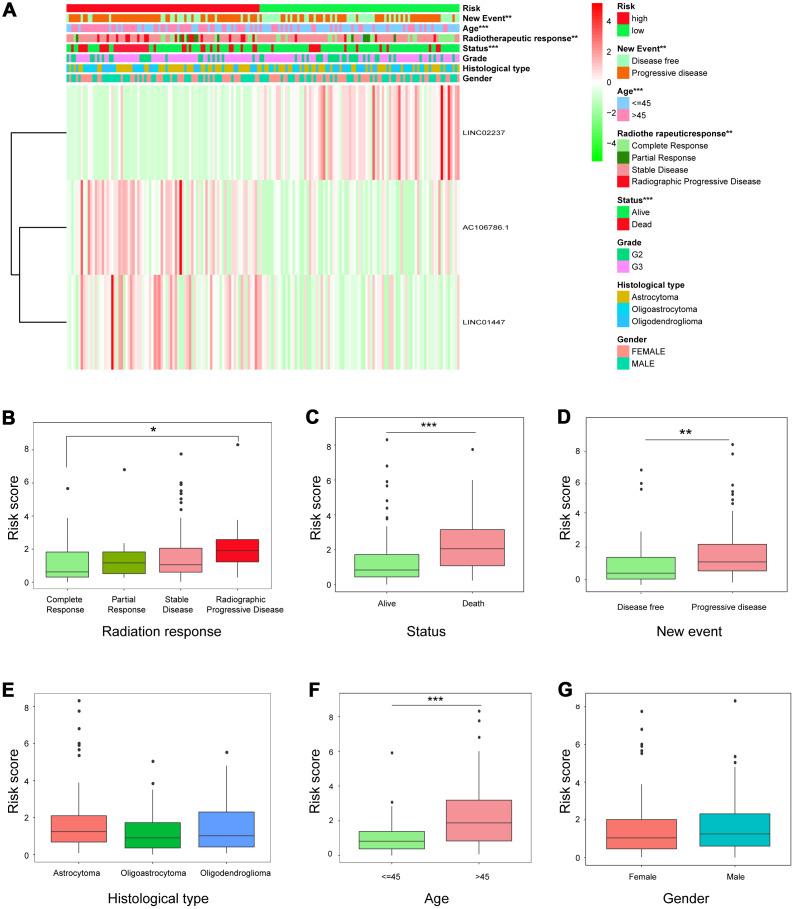
**Relationship between risk score and clinicopathological features.** (**A**) Heatmap showing the expression of the three lncRNAs in low- and high-risk groups; distributions of clinicopathological features were compared between the low- and high-risk groups. (**B**–**G**) Risk score distributions when patients were stratified by (**B**) radiation response, (**C**) status, (**D**) new event, (**E**) histological type, (**F**) age, and (**G**) gender.

Expression of the three-lncRNA signature in high-risk and low-risk patients is shown in Figure 5A. Significant differences were observed between the high- and low-risk groups with respect to radiotherapeutic response (*P*<0.01), new event incidence (*P*<0.01), age (*P*<0.001), and status (*P*<0.001). Patients in the low-risk group were more likely to exhibit CR or PR after radiotherapy, and fewer low-risk patients died or experienced disease progression.

Individual associations between risk score and clinicopathological features were also identified. Risk scores differed between the groups when patients were stratified by radiotherapeutic response, new event incidence, age, and status, but not by histological type and gender (Figure 5B–5G), indicating that the risk scores calculated with the signatures could accurately predict radiotherapy outcomes, survival, and clinicopathological features in LGG patients.

**Figure 6:**
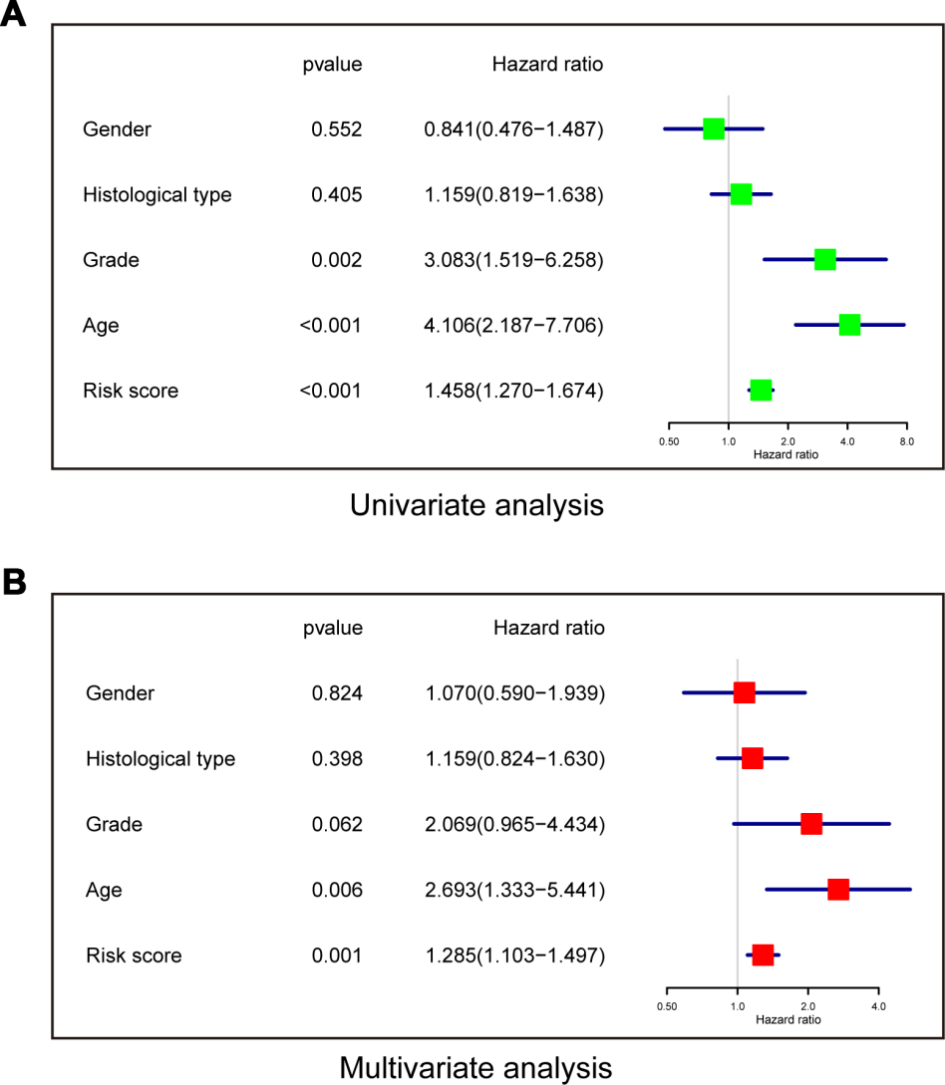
**The risk model is an independent prognostic indicator for overall survival among LGG patients after radiotherapy.** (**A**) Univariate analysis. (**B**) Multivariate Cox regression.

Univariate and multivariate Cox regression analyses were performed to determine whether the risk model was an independent prognostic indicator. In the univariate analysis, risk score, grade, and age were all correlated with OS (Figure 6A). In the multivariate analysis including these factors, risk score and age remained significantly associated with OS (Figure 6B). These results demonstrated that the risk score derived from the three-lncRNA signature could independently predict prognosis in LGG patients who received radiotherapy.

### Radiation resistance-associated genes and signal pathways were up-regulated in the high-risk group

**Figure 7:**
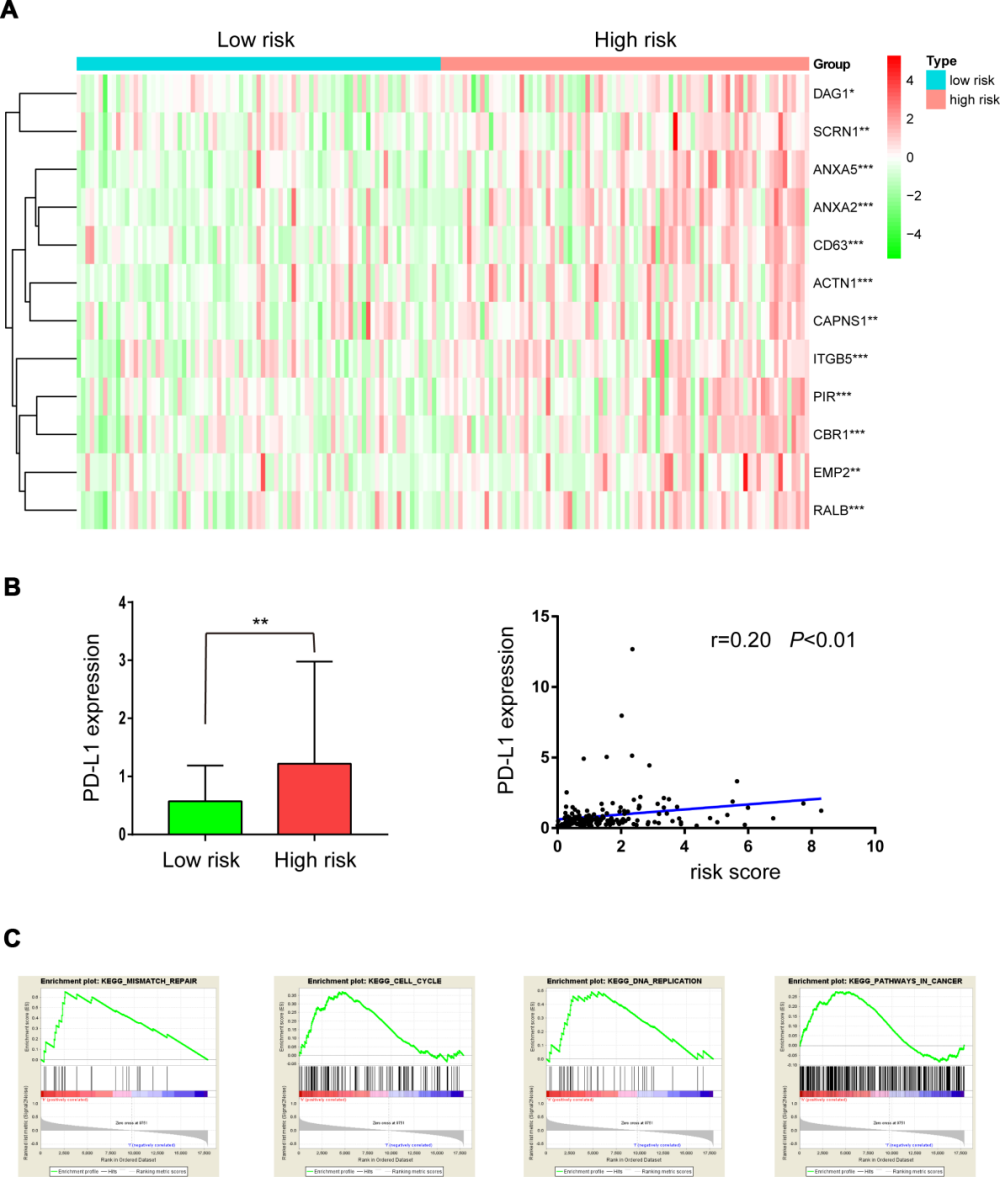
**Expression of radioresistant genes and GSEA enrichment analysis in low- and high-risk groups.** (**A**) Heatmap showing the expression of 12 radioresistant genes that were up-regulated in the high-risk group; (**B**) PD-L1 expression in low- and high-risk groups; (**C**) GSEA enrichment in low- and high-risk groups.

A “31-gene signature” has been reported for predicting outcomes after radiotherapy in LGG patients. Up-regulation of 19 of these genes was associated with radiation resistance [10, 11]. Here, we investigated whether these genes were up-regulated in the high-risk group. As shown in Figure 7A, 12 of 19 radiation resistance-associated genes were overexpressed in the high-risk group compared to the low-risk group.

In a previous study, PD-L1 was overexpressed in radiation-resistant cell lines, and tumors with high PD-L1 expression had high failure rates following radiotherapy. Here, as shown in Figure 7B, PD-L1 was overexpressed in the high-risk group and was positively correlated with risk score (r=0.20, *P*<0.01), suggesting that PD-L1 inhibitors might benefit high-risk LGG patients receiving radiation treatment.

GSEA enrichment analysis was used to identify pathways enriched in high risk patients compared to low risk patients. Gene sets (Fig 7C) related to mismatch repair, cell cycle, DNA replication, and pathways in cancer, all of which may contribute to radiation resistance, were differentially enriched in high risk patients.

### Downregulation of LINC01447 or AC106786.1 sensitized low-grade glioma HS683 cells to irradiation

**Figure 8:**
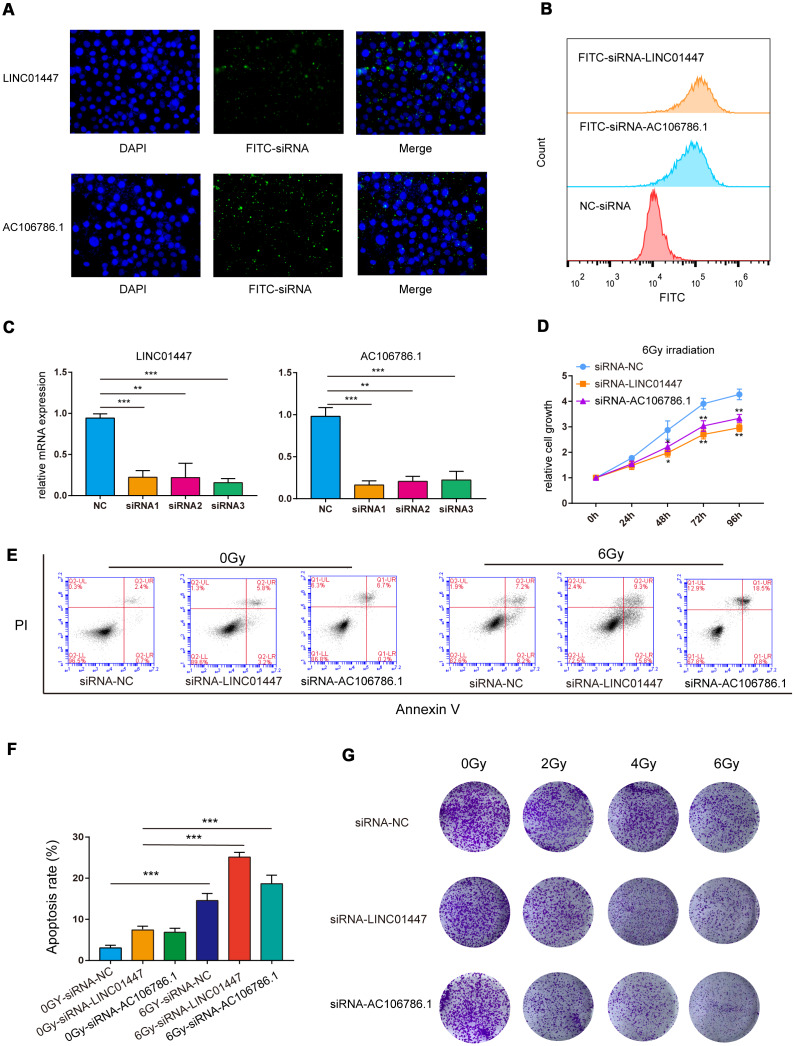
**Downregulation of LINC01447 or AC106786.1 enhanced radiosensitivity in low-grade glioma cells.** (**A**) Representative fluorescence microscope images of HS683 cells treated with FITC-siRNA-LINC01447 or FITC-siRNA-AC106786.1: blue = DAPI; green = FITC-siRNA. (**B**) Representative flow cytometry histograms showing cellular uptake of FITC-siRNA-LINC01447 (orange), FITC-siRNA-AC106786.1 (blue), and NC-siRNA (red). (**C**) Silencing efficiency was evaluated using real-time PCR. (**D**) CCK8 assays were used to investigate the roles of LINC01447 and AC106786.1 in HS683 cell proliferation after irradiation. (**E**, **F**) Apoptotic cells were detected by Annexin V-FITC and PI staining. Apoptosis ratio was calculated by adding early and late apoptosis percentages. (**G**) Colony formation efficiency was used to evaluate the radiosensitivity of treated HS683 cells. *P < 0.05; **P < 0.01; ***P < 0.001.

The predictive model consisted of LINC02237, AC106786.1 and LINC01447, and overexpression of LINC01447 and AC106786.1 was associated with decreased OS and PFS times. To further validate the effects of these lncRNAs on radiation response, HS683 low-grade glioma cells were transfected with LINC01447-siRNA or AC106786.1-siRNA (Figure 8A, 8B), and then exposed to single radiation doses of 0, 2, 4, or 6 Gy. Cell Counting Kit-8 (CCK8), apoptosis, and colony formation assays were performed to determine radiosensitivity.

The siRNAs successfully downregulated expression of LINC01447 and AC106786.1 (Figure 8C). The CCK-8 assay demonstrated that cell viability decreased 48, 72, and 96 hours after 6 Gy radiation in HS683 transfected with either LINC01447-siRNA or AC106786.1-siRNA (Figure 8D). Next, flow cytometry was performed to determine whether this lncRNA-mediated decrease in radiation resistance was due to apoptosis. Apoptosis rates increased in both LINC01447-siRNA and AC106786.1-siRNA cells 48 h after treatment with 6 Gy radiation compared to control cells (Figure 8E, 8F). Colony formation assays showed that downregulation of LINC01447 or AC106786.1 inhibited survival and foci formation in cells exposed to IR (Figure 8G). Taken together, these results show that downregulation of LINC01447 or AC106786.1 enhanced the radiosensitivity of low-grade glioma cells.

## DISCUSSION

Radiotherapy is often used to control LGG, but LGG patients with resistance to radiation do not benefit from such treatment and suffer from adverse effects. It is therefore important to identify reliable biomarkers for predicting response to radiation in LGG patients. Comprehensive mRNA expression analysis has been used to identify radiosensitivity-related factors in breast, colorectal, and nasopharyngeal, head, and neck cancer [12–15]. Here, we used high-throughput lncRNA profiling data to determine the utility of lncRNAs as prognostic biomarkers for predicting patient outcomes after radiotherapy.

Previous studies have demonstrated that lncRNAs impact radioresistance through various mechanisms, including reversal of cell cycle arrest, DNA damage, apoptosis, epithelial–mesenchymal transition (EMT), MAPK signaling pathway, and autophagy [16]. In this study, we identified 37 differentially expressed lncRNAs that were associated with radiation response. Most of them have not been reported previously in cancer. Further investigation of their potential target mRNAs demonstrated that they might contribute to radioresistance in LGG patients via the PI3K-Akt signaling pathway, MAPK signaling pathway, activation of cell proliferation, and inhibition of apoptotic processes and DNA damage response; these mechanisms are consistent with findings on resistance to radiotherapy in other tumors [17, 18].

Next, we evaluated whether the identified lncRNAs could predict LGG patient prognosis after radiotherapy. A three-lncRNA signature was constructed based on univariate and multivariate Cox regression analyses and was used to separate LGG patients who received radiotherapy into high- and low-risk groups. Indeed, patients in the low-risk group responded well to radiotherapy as indicated by fewer deaths and lower incidence of disease progression. In contrast, high-risk patients were likely to experience resistance to radiotherapy, disease progression, and poor prognosis. Combination therapies might therefore improve outcomes in high risk patients. For example, preclinical and clinical studies show that PD-L1 blockade in combination with radiotherapy results in stronger antitumor effects. In this study, PD-L1 was overexpressed in the high-risk group and was positively correlated with risk score. This indicates that PD-L1 blockade in combination with radiotherapy might benefit patients with high risk scores as well.

Recently, a 31-gene signature that predicts radiation sensitivity and clinical outcomes in invasive breast carcinoma, lower-grade glioma, and head and neck cancer was identified using integrative meta-analysis of published microarray data from TCGA for NCI-60 cancer cells [11, 12, 19]. Among those 31 genes, 19 were associated with resistance to radiation. Here, 12 of those 19 radiation resistance-associated genes were overexpressed in the high risk group, further confirming that our three-lncRNA signature can successfully differentiate between patients who are sensitive and resistant to radiotherapy. The mechanism of radiation resistance is complex and multigenic. Although there is no evidence of overlapping with existing 31-gene signature in our lncRNAs and report genes, the underlying pathway regulated by these lncRNAs, like PI3K-Akt signaling pathway, MAPK signaling pathway, activation of cell proliferation, inhibition of apoptotic processes and DNA damage response, are consistent with findings on resistance to radiotherapy in other tumors [17, 18].

PD-L1 has been identified as a promising target for immune therapy and as a candidate biomarker of treatment failure following radiation in head and neck cancer; the radiotherapy failure rate in tumors with high PD-L1 expression was 60% compared to 20% in the low PD-L1 expression group [20]. Here, PD-L1 was overexpressed in the high-risk group and was positively correlated with risk score in LGG patients, suggesting that it may contribute to radiation resistance in high-risk patients.

Next-generation sequencing has identified thousands of lncRNAs for which aberrant expression is associated with various cancer types. Among the few that have been functionally characterized, several have been linked to malignant transformation. Notably, these lncRNAs play critical roles in gene regulation and various processes related to tumor progression, including proliferation, survival, invasion, and migration [21]. LncRNAs primarily interact with mRNA, miRNA, DNA, and proteins, which in turn regulate gene expression at the epigenetic, transcriptional, post-transcriptional, translational, and post-translational levels in a variety of ways [22]. LncRNAs may therefore prove to be significant biomarkers for cancer prognosis, diagnosis, and even prediction of therapeutic outcomes. The development of multi-gene risk models may improve genetic cancer risk assessment and help to improve clinical decisions, especially in patients with high PD-L1 who often experience failures after radiotherapy.

In summary, we examined the role of various lncRNAs in low-grade glioma radiation sensitivity and constructed a risk-score model based on three lncRNAs to predict outcomes in LGG patients following radiotherapy. Additional prospective clinical studies should be conducted to validate the predictive value of these biomarkers.

## MATERIALS AND METHODS

### Data acquisition and processing

Gene expression data and clinical information were obtained from The Cancer Genome Atlas database (https://tcga-data.nci.nih.gov/tcga/). Data was downloaded for a total of 167 LGG patients for which radiation response information was available, including 32 patients with complete response, 11 with partial response, 103 with stable disease, and 21 with radiographic progressive disease after receiving radiotherapy (Workflow Type: HTSeq-FPKM).

### Identification of differentially expressed lncRNAs (DElncRNAs)

Patients with a complete response or partial response after radiotherapy were assigned to the radiosensitive group, while those with stable disease or radiographic progressive disease were assigned to the radioresistant group. Next, lncRNAs that were differentially expressed between the radiosensitive and radioresistant groups were identified using the edgeR R package. DElncRNAs with |log_2_ fold change|>1 and a *P* value < 0.05 were considered for subsequent analysis. The Volcano map package for R was used to describe the DElncRNAs.

### Weighted correlation network analysis (WGCNA)

To find the potential target genes associated with DElncRNAs, we constructed a co-expression network using the R package WGCNA. The soft thresholding power was set to 6 to produce a weighted network. A lncRNA-mRNA regulator network was constructed using edge weights > 0.5, and lncRNA-mRNA connections were further visualized by Cytoscape.

### Constitution of a risk model

Statistically significant DElncRNAs in univariate Cox regression analysis were used in multivariate Cox regression to determine coefficients, and the risk-score formula was defined as follows:

$$\text{risk}\;\text{score}=\sum_{\text{i}=1}^{\text{N}}\left(Exp_{i}*Coe_{i}\right)$$

where N=3, *Exp_i_* was the expression value for each of the 3 lncRNAs and *Coe_i_* was the corresponding coefficient from multivariate Cox regression analysis.

### Survival analysis

OS and PFS were compared between the high and low DElncRNA expression groups and between the high- and low-risk groups via Kaplan-Meier analysis using the Survival and Survminer package in R. Univariate Cox analysis was performed to identify potential prognostic factors, and multivariate Cox analysis was used to evaluate risk score as an independent risk factor for PFS and OS in LGG patients who received radiotherapy. A receiver operating characteristic (ROC) curve was generated to validate the accuracy of the risk model in predicting patient OS and PFS using the survivalROC R package.

### Functional enrichment analysis

The “ClueGO” app in Cytoscape software was used to analyze Kyoto Encyclopedia of Genes and Genomes (KEGG) pathways [19]. Gene ontology (GO) analysis was carried out using DAVID, a website with gene annotation and integrated discovery functions, and visualized using the GOplot R package. GO and KEGG enrichment analyses were based on threshold *p*-value < 0.05 and *q*-value < 0.05.

GSEA was performed to identify a set of genes with significantly differential expression between the high and low-risk groups using enrichment data from the MSigDB Collection (c2.cp.kegg.v7.0.symbols.gmt). Gene set permutations were performed 1000 times for each analysis. The phenotype label was used as a risk score.

### Cell culture

Low-grade glioma HS683 cells were purchased from Shanghai Genechem Co., Ltd. HS683 cells were cultured in DMEM (Invitrogen, Carlsbad, CA, USA) with 10% fetal bovine serum (HyClone, Logan, UT, USA).

### siRNAs and *in* *vitro* transfection

Three siRNA sequences targeting LINC01447 or AC106786.1 were synthesized by Guangzhou Rui Bo Biological Technology. Cells were transfected with siRNAs using Lipofectamine RNAiMAX transfection reagent (Invitrogen) based on the manufacturer’s instructions. Briefly, cells were seeded to a confluence of 50-60% and transfected with siRNAs (50 nM). Control cells were treated with NC-siRNA. The siRNA sequences were as follows:

LINC01447 siRNA1: Sense, 5’-ACUUCUACUCAAU AGAACCTT-3’,

Antisense, 5’-GGUUCUAUUGAGUAGAAGUTT-3’;

LINC01447 siRNA2: Sense, 5’-AGAAUGAGGCGGA GUUUGGTT-3’,

Antisense, 5’-CCAAACUCCGCCUCAUUCUTT-3’;

LINC01447 siRNA3: Sense, 5’-AAUCUUCAUGGAU CUCUUCTT-3’,

Antisense, 5’-GAAGAGAUCCAUGAAGAUUTT-3’;

AC106786.1 siRNA1: Sense, 5’-UCAGAAAAUCUAU UUUGUGTT-3’,

Antisense, 5’-CACAAAUAGAUUUUCUGATT-3’;

AC106786.1 siRNA2: Sense, 5’-AACAUUUUCGGU CUAACUCTT -3’,

Antisense, 5’-GAGUUAGACCGAAAAUGUUTT-3’;

AC106786.1 siRNA3: Sense, 5’-AAUCUAUCCAACA AUGACGTT-3’,

Antisense, 5’-CGUCAUUGUUGGAUAGAUUTT-3’;

NC-siRNA: Sense, 5’- UUCUCCGAACGUGUCACG UTT-3’,

Antisense, 5’- ACGUGACACGUUCGGAGAATT-3’.

### Apoptosis assay

Cells transfected with siRNA-LINC01447 or siRNA-AC106786.1 were seeded in 6-well plates and irradiated with 6 Gy of X-rays; they were then incubated for 48 h. For apoptosis assays, cells were trypsinized, washed, resuspended in 200 μL binding buffer, and then analyzed for apoptosis by double staining with 5 μL annexin V and 5 μL propidium iodide (Beyotime Biotechnology, China) and using a BD Accur™ C6 Flow Cytometer (BD Inc, Piscataway, NJ).

### CCK-8 assay

For cell proliferation assays, 800 HS683 cells were seeded in 96-well plates after transfection and irradiated with 6 Gy of X-rays. CCK8 reagent (Beyotime Biotechnology, China) was added to each well to measure the number of viable cells after 0, 24, 48, 72, and 96 h. Optical density was measured at a wavelength of 450 nm (OD450).

### Colony formation assays

For colony formation assays, 5000 HS683 cells were seeded in 6-well plates after transfection with siRNA-LINC01447 or siRNA-AC106786.1; they were then incubated for 24 h. Cells were treated with radiation doses of 2, 4, and 6 Gy. Approximately 14 days later, colonies were stained with 0.5% crystal violet and pictures were taken.

### Real-Time PCR

Total mRNA was isolated from HS683 cells transferred with siRNA-LINC01447, siRNA-AC106786.1 or NC-siRNA using Trizol (Invitrogen, UK), and cDNA was synthesized from 100 ng of total RNA using the PrimeScript™ RT reagent Kit with gDNA Eraser (TaKaRa, Japan). Q-PCR was conducted with GoTaq qPCR Master Mix (Promega, USA) and Applied Biosystems 7500 Real-Time PCR Systems. All samples were normalized to β-actin mRNA levels. The primer sequences were as follows:

LINC01447: Forward: 5’-CTCTACCAATCAGCAGG ATGTG-3’;

Reverse: 5’-AAGTGAGCAGCAGCAAGATT-3’.

AC106786.1: Forward: 5’-CGGCACAATCTCTAGGA CTCT-3’;

Reverse: 5’-ACCACCAACCTTCCTATCTACC-3’.

β-actin: Forward: 5’- CATGTACGTTGCTATCCAGG C -3’;

Reverse: 5’- CTCCTTAATGTCACGCACGAT -3’.

## Supplementary Material

Supplementary Table
