# Supplementary material for: A three-lncRNA signature predicts clinical outcomes in low-grade glioma patients after radiotherapy
Source: Aging (Albany NY). 2020 May 26;12(10):9188–204. doi: 10.18632/aging.103189 (PMC7288909; doi:10.18632/aging.103189)
Supplement: Supplementary Table [file aging-12-103189-s001..pdf]

SUPPLEMENTARY TABLE

Supplementary Table 1. 13 up-regulated lncRNAs and 24 down-regulated lncRNAs in radioresistant groups.

lncRNA	logFC	logCPM	PValue	FDR
AC004832.1	-6.5846	4.90548	7.74E-32	6.56E-28
AL157831.2	-6.08761	4.514612	3.66E-30	1.55E-26
AC023796.1	-7.49029	4.462543	1.92E-23	5.44E-20
AL078605.1	-2.58287	5.363397	1.39E-20	2.94E-17
AC074351.1	-2.14218	5.653697	1.12E-13	1.90E-10
AC084816.1	-2.69039	3.368313	7.68E-13	1.08E-09
AC000061.1	-4.78751	2.166427	2.20E-12	2.67E-09
AC023886.1	-2.10256	3.70055	7.94E-10	8.41E-07
AL133372.2	-3.36141	2.164238	1.15E-09	9.94E-07
AL137005.1	-3.42876	3.038507	1.17E-09	9.94E-07
AC004870.2	-2.3616	3.710813	2.45E-09	1.89E-06
AC073324.2	-2.5801	5.087589	6.55E-09	4.62E-06
LINC02237	-1.79369	2.355266	2.55E-07	0.000166
LINC01479	4.373595	4.257885	4.55E-07	0.000273
AC002384.1	-3.02415	2.035558	4.83E-07	0.000273
FAM30A	-2.00246	3.185267	5.85E-07	0.00031
AC046168.2	-1.05344	6.818276	1.16E-06	0.000579
MUC2	3.876152	4.10967	1.36E-06	0.000642
AP000221.1	-1.55528	3.12966	1.53E-06	0.000684
AC022079.1	-1.26838	3.289331	3.29E-06	0.001396
KC6	3.413798	3.182956	4.12E-06	0.001664
GS1-24F4.2	-1.1294	4.07537	6.00E-06	0.00231
AC022098.2	3.279784	3.071052	1.21E-05	0.004371
LINC01163	-1.3317	3.225518	1.24E-05	0.004371
AC106786.1	-1.50296	3.332785	1.68E-05	0.005714
AC087241.4	-1.51953	2.153561	2.51E-05	0.008182
AP003083.1	-1.285	5.43157	3.42E-05	0.010731
AC020659.1	1.786279	4.027274	4.18E-05	0.012232
AL133415.1	1.190541	4.741589	6.98E-05	0.019717
DISC1FP1	-1.64751	2.27533	7.98E-05	0.021447
AC023905.1	3.127208	2.10345	8.10E-05	0.021447
AC022498.1	1.522007	3.866576	9.02E-05	0.023175
OTX2-AS1	3.50496	3.271446	0.000106	0.025632
LINC01447	2.159006	5.885161	0.000157	0.036866
AC092343.1	2.3475	2.50476	0.000186	0.042157
LINC01239	1.585229	3.873012	0.000189	0.042157
AC112487.1	1.523493	3.6163	0.000217	0.046079
